# Effects of pollution on adolescent mental health: a systematic review protocol

**DOI:** 10.1186/s13643-021-01639-z

**Published:** 2021-03-27

**Authors:** Linda C. Theron, Yael Abreu-Villaça, Marcus Augusto-Oliveira, Caroline H. Brennan, Maria Elena Crespo-Lopez, Gabriela de Paula Arrifano, Lilah Glazer, Liyuan Lin, Isabelle Mareschal, Luke Sartori, Liesl Stieger, Andres Trotta, Kristin Hadfield

**Affiliations:** 1 Department of Educational Psychology, University of Pretoria, Pretoria, South Africa; 2 Departamento de Ciências Fisiológicas, Instituto de Biologia Roberto Alcantara Gomes, Universidade do Estado do Rio de Janeiro, Rio de Janeiro, Brazil; 3 Laboratory of Molecular Pharmacology, Institute of Biological Sciences, Federal University of Pará, Belém, Brazil; 4 Department of Biological and Experimental Psychology, Queen Mary University of London, London, UK; 5 Nanchang Joint Programme, School of Biological and Chemical Sciences (SBCS), Queen Mary University of London, London, UK; 6 Education Library, University of Pretoria, Pretoria, South Africa; 7 Institute of Collective Health, National University of Lanús, Remedios de Escalada, Argentina; 8 Trinity Centre for Global Health, Trinity College Dublin, Dublin, Ireland

**Keywords:** Narrative synthesis, Mental disorder, Pollutants, Pollution-associated risks, Resilience, Systematic review protocol, Adolescent, Mental health

## Abstract

**Background:**

Whilst there is little uncertainty about the deleterious impact of pollution on human and planetary health, pollution’s impact on adolescent mental health is less well understood. This is particularly true for young people in underdeveloped and developing world contexts, about whom research is generally lacking. Furthermore, although adolescent resilience continues to be a research priority, little attention has been paid to adolescent pathways of resilience in the face or aftermath of pollution exposure. The objective of this study will be to examine the associations between pollution and mental health in 10- to 24-year-olds (i.e. adolescents).

**Methods:**

We designed and registered a study protocol for a systematic review of studies which link pollution and mental health in adolescents. We will include observational studies (e.g. cohort, case-control, time series analyses) that assess the associations between exposure to any form of pollution and the mental health of 10- to 24-year-olds. The primary outcome will be symptoms associated with neurodevelopmental disorders; disruptive, impulse-control, and conduct disorders; depressive disorders; anxiety disorders; substance disorders; and schizophrenia. No secondary outcomes will be considered. Literature searches will be conducted in multiple electronic databases (from inception onwards), including PubMed, MEDLINE, SCOPUS, Web of Science, CINAHL, PsycINFO, SciELO, ERIC, and Africa-Wide. Two investigators will independently screen all citations, full-text articles, and abstract data. The methodological quality (or bias) of included studies will be appraised using appropriate tools. We will provide a narrative synthesis of the evidence.

**Discussion:**

This systematic review will evaluate the evidence on the associations between pollution and the mental health of 10- to 24-year-olds. Our findings will be of potential interest to multiple audiences (including adolescent patients/clients, their families, caregivers, healthcare professionals, scientists, and policy makers) and could be used to develop prevention and intervention strategies as well as focus future research. Results will be published in a peer-reviewed journal.

**Systematic review registration:**

PROSPERO CRD42020176664

**Supplementary Information:**

The online version contains supplementary material available at 10.1186/s13643-021-01639-z.

## Background

Across the globe, human activity has resulted in widespread emissions that are harmful to the earth and its inhabitants [1]. Harmful emissions pollute the air, water, and/or soil [2] via substances—including plastics, heavy metals, pesticides, building materials, antibiotics, and synthetic hormones—heat, vibrations, or noise [3, 4]. Pollution’s harmful physical health sequelae are well-recognized [5–7].

In comparison, understanding of the mental health effects of pollution is less robust [8–10], particularly when it comes to impacts on young people [11, 12]. Even when studies have included young people’s mental health, there is almost no focus on adolescents (i.e. young people aged 10 to 24 [13]) and mental health is restricted to neurodevelopmental challenges (i.e. autism spectrum disorder [ASD], attention deficit hyperactivity disorder [ADHD], impaired cognitive functioning/learning capacity). For instance, a systematic review of 63 articles published between 2000 and 2018 investigated six physical and neurodevelopmental outcomes among infants and children exposed to air-borne pollutants associated with fossil fuel combustion [14]. The review provided unequivocal evidence that exposure to air pollution is deleterious to the physical and neurodevelopmental health of infants and children (mostly < 10 years). In comparison, only one of the articles included in the review specified neurodevelopmental outcomes for adolescents (i.e. hyperactivity and/or inattention at age 15). A subsequent narrative review of 134 articles relating to pollution and mental health by Ventriglio and colleagues [15] similarly included little on adolescent mental health. The review, which was not systematic, reported neurodevelopmental disorders (including ADHD and ASD) and cognitive deficits among children (generally younger than 10) exposed to air, light, or noise pollution; heavy metals; and/or pesticides. This review made little reference to mental illness that was not neurodevelopmental (such as depression or anxiety) [15]. There was a single mention of increased ‘psychiatric conditions’ following children’s exposure to ultrafine particles and one of ‘a mental disorder’ following a 16-year-old female’s exposure to mercury. Another example of the limited attention to pollution effects on adolescent mental health is the review by Freire and Koifman [16]. These authors conducted a systematic review of 25 studies that investigated pesticide exposure and depression/suicide. Eight of the 25 reviewed studies reported samples that included adolescents (10- to 24-year-olds [13]). Even so, there were no adolescent-specific conclusions relating to the overall finding that there was a limited evidence base linking pesticide exposure and depression or suicide.

The under-attention to pollution’s potential mental health effects in adolescence is problematic, not least because much of the global burden of disease is attributable to mental illness [17]. Half of all mental disorders are thought to have commenced by early adolescence [18]. Such early onset is associated with 10 times the expense of disorders with later onset [19]. Moreover, poor adolescent mental health predicts constrained development along with long-term diminished cognitive, psychological, and behavioural capacities [20, 21]. Furthermore, adolescence is a time of substantial personal change that impacts how adolescents interact with the world (and, therefore, pollutants). These concerns beg systematic attention to the mental health of adolescents, with specific consideration of those who are exposed to any form of pollution (i.e. air, water, and/or soil—including plastics, heavy metals, pesticides, building materials, antibiotics, and synthetic hormones —and heat, vibrations, or noise). This attention must be inclusive of adolescents in low- and middle-income countries (LMICs), given that 85% of the world’s young people reside in LMICs [22] and the understanding that they may be disproportionately impacted by exposure to pollution [1].

Consideration of adolescent mental health should not omit the factors and processes that enable or sustain mental health [23, 24]. Whilst young people’s capacity to maintain positive mental health despite exposure to risk is well-researched [25], there is limited understanding of the resilience processes that protect adolescent mental health specifically during and/or following exposure to environmental pollution [26]. Given the paucity of mental health services and adequately trained staff, particularly in LMICs [17, 27], leveraging the factors and processes that support mental health resilience could forestall the need for mental health services. Because resilience processes are multi-systemic and sensitive to developmental, situational, and cultural determinants [28], it will be important to ascertain what facilitates adolescent resilience to pollution exposure across diverse contexts and highlight contextually relevant resilience-enablers.

Taken together, the abovementioned concerns prompt our interest in what is currently known about the associations between pollution and adolescent mental health worldwide. This interest is framed by a social-ecological perspective of resilience, i.e. the understanding that positive human adaptation to significant risk, such as pollution, is a dynamic and contextually responsive process [29]. Accordingly, the aim of this study will be to evaluate the associations between pollution and adolescent mental health across diverse contexts and throughout their development. To this end, the proposed systematic review will answer the following questions:

1. What is the association between pollution exposure (type/level) and symptoms of adolescent mental illness (e.g. symptoms associated with neurodevelopmental disorders like autism, attention deficit, and/or hyperactivity disorder; conduct disorders; depression, anxiety, and substance disorders)?
2. In what ways, if any, are these associations different across adolescent development and diverse geographical contexts?
3. In instances where minimal symptoms of adolescent mental illness are reported, which protective factors, if any, are/could be associated with adolescent mental-health resilience to pollution exposure?
4. What clinical and/or study methodological characteristics might explain any heterogeneity in results?

## Methods

The present study protocol is being reported in accordance with the reporting guidance provided in the Preferred Reporting Items for Systematic Reviews and Meta-Analyses Protocols (PRISMA-P) statement [30, 31] (see PRISMA-P checklist in Supplemental File 1). This protocol has been registered within the International Prospective Register of Systematic Reviews (PROSPERO) database (registration ID: CRD42020176664).

### Eligibility criteria

Studies will be selected based on the following criteria.

#### Study design

Only original, human studies will be included. Any observational study (i.e. study in which outcomes are not manipulated) is eligible, except for qualitative, in silico (i.e. computer-simulated), or intervention ones (e.g. interventions to limit adolescent exposure to pollution or improve adolescent mental health). The previously mentioned studies are unlikely to provide evidence that addresses the proposed review’s purpose and thus we have limited our scope to observational studies.

#### Participants

We will include studies of people aged 10 to 24 years from any country. According to Sawyer et al. [13], this age range constitutes adolescence. Traditionally, adolescence spanned 10 to 19 years [18]. The decision to further include 20- to 24-year-olds in the definition of adolescence reflects recent arguments for adolescence to include those who would traditionally have been considered emerging adults [13], given how dependence on parents has been extended by modern societies’ valuing of post-school education/training and global trends of youth unemployment. Our exclusive focus on adolescents relates to adolescence being a sensitive developmental period when half of all mental health problems develop and manifest [18]. Furthermore, adolescence is a time of substantial personal change that impacts how adolescents interact with the world (and, therefore, pollutants).

#### Exposures

To be included, studies must investigate adolescent exposure to pollution. Following the European Union’s definition [4], pollution is understood as ‘the direct or indirect introduction, as a result of human activity, of substances, vibrations, heat or noise into air, water or land which may be harmful to human health or the quality of the environment’ (p. 6). Studies that make tangential reference to pollution (e.g. only in the introduction or recommendations) and do not measure it in some way will be excluded. The same applies to studies in which solvents or pesticides are purposefully used to self-harm (e.g. substance abuse or suicide). Studies reporting exposure to natural (rather than anthropogenic) sources of heat will also be excluded.

#### Outcomes

To be included, studies must investigate the mental health of adolescents exposed to pollution. For adolescents, mental health implies no or limited indication of (i) neurodevelopmental disorders; (ii) disruptive, impulse-control, and conduct disorders; (iii) depressive disorders; (iv) anxiety disorders; or (v) substance disorders [32]. Lee et al. [19] also include schizophrenia in typical adolescent-onset disorders. All these disorder groupings are recognized by the 5th edition of the Diagnostic and Statistical Manual of Mental Disorders (DSM-5^TR^) [33]. The DSM-5^TR^ guides practitioner support of mental health. Studies that did not measure mental health outcomes (e.g. through a clinical interview, scale/checklist, self- or adult-report) will be excluded.

#### Report characteristics

Peer-reviewed, indexed journal articles (published and pre-print, online) will be included. Reports could be published in any language and at any time. In instances where the same data set is reported in multiple articles, the article that provides the clearest evidence of pollution associations with adolescent mental health will be included.

### Information sources

Peer-reviewed articles, published on or before 10 April 2020, will be retrieved using the following databases: Africa-Wide, CINAHL, ERIC, PsycARTICLES, and PsycINFO (all via EBSCOhost platform); MEDLINE (via Web of Science Clarivate Analytics); PubMed; Scopus (which includes contents of Embase); Web of Science Core Collection; and SciELO Citation Index. The database search will be supplemented by a manual search of the reference lists of well-cited articles identified in the database search and by contact with included study authors. The reviewer team (of which researchers from the Global South comprise the majority) will be sensitive to the inclusion of indexed Global South studies given their historic under-representation in scholarly literature [34]. Following Bellefontaine and Lee [35], grey literature will only be included should the aforementioned information sources show that the published literature is limited.

### Search strategy

The search strategy (see Supplemental File 2) was developed by author LS1, a librarian working in South Africa, and tested by LS2, a research assistant working in the UK. No date or language limits will be imposed, but the search will be limited to scholarly/peer-reviewed journal publications. The terms listed in the search strategy will be searched for in the title, abstract, and topic fields (for the databases that allow such delimiters).

### Study records

#### Data management

Articles meeting the search strategy will be populated into EndNote and screened for duplicates. Once duplicates have been removed, the records will be exported to Zotero, a software programme that allows citations to be formulated as title and abstract.

#### Selection process

The first 50 citations (title and/or abstract) will be independently screened by 11 of the review authors (LT, YAV, CB, MECL, GPA, MAO, LG, LL, IM, AT, KH) and results compared via consensus discussion (see Saldana [36]). This will support reviewer familiarity with the inclusion/exclusion criteria and calibrate application of the criteria. Thereafter, the remaining citations will be divided into five sets. Each set will be independently screened by at least 2 reviewers. Titles/abstracts that meet the inclusion criteria—as well as those where there is uncertainty—will be selected for a full-text review by LL and KH. Consensus discussions (see Saldana [36]) will again be used to resolve any disagreements. Should consensus not be reached, LT will arbitrate.

#### Data collection process

Data extraction will be guided by a data-charting form that will be developed by LT and KH and calibrated by all reviewers (using 10 of the eligible articles). The data-charting form will correspond to the items for which data will be sought (see the ‘Data items’ section). At least 2 reviewers will independently extract data from each eligible article. In instances of reviewer disagreement about extracted data, LT and KH will arbitrate. If necessary, study authors will be approached to clarify uncertainties.

### Data items

Data will be extracted as follows.

#### Study design

We will extract the type of design and methods, sample size and type (e.g. random or purposive), population sampled from, data collection instruments (including those to assess pollutant exposure or measure mental health), study duration, data collection dates, ethical procedures, and study funding (if any).

#### Participants

We will extract detail about age (e.g. age range, average age) and, where possible, other demographic detail (e.g. sex/gender, race/ethnicity, nationality, urban/rural/other location, education, socio-economic status).

#### Exposures

We will extract data relating to direct exposure to air-, water-, or land-based substances, vibrations, heat, or noise, as well as duration and frequency of direct exposure. We will also extract data relating to indirect exposure to air-, water-, or land-based substances, vibrations, heat, or noise, as well as duration and frequency of the indirect exposure.

#### Mental health impacts

We will extract data relating to symptoms of (i) neurodevelopmental disorders; (ii) disruptive, impulse-control, and conduct disorders; (iii) depressive disorders; (iv) anxiety disorders; (v) substance disorders; or (vi) schizophrenia spectrum disorders. Where possible, we will distinguish between acute (i.e. requiring institutionalization or hospitalization) and other impacts (i.e. any non-in-patient treatment, such as medication or counselling). In instances where journal articles specify related DSM-5^TM^ or ICD codes, these will be recorded. Should publications report evidence of causal mechanisms for pollutants’ mental health impacts, then we will extract these too.

#### Factors or processes that protect mental health

Following Ungar and Theron [28], we will extract data relating to biological, psychological, social, structural, or ecological factors or processes that are reported to be associated with mental health resilience.

#### Publication details

We will extract detail about whether the article is open or closed access, the existence of a study protocol, and the language(s) of publication.

### Outcomes and prioritization

The primary outcomes will be evidence (or not) of symptoms of (i) neurodevelopmental disorders; (ii) disruptive, impulse-control, and conduct disorders; (iii) depressive disorders; (iv) anxiety or post-traumatic stress disorders; (v) substance disorders; or (vi) schizophrenia spectrum disorders. Each of the aforementioned constitutes a cluster of disorders. For example, as per the DSM-5^TM^, depressive disorders comprise 8 disorders (e.g. disruptive mood dysregulation disorder, major depressive disorder, persistent depressive disorder) [33]. Using DSM-5^TM^ diagnostic criteria, each of these can be further specified and assigned a specific DSM-5^TM^ code. For example, major depressive disorders can present as a single or recurrent episode that is mild, moderate, severe, with psychotic features, in partial remission, in full remission, or unspecified [33]. Given that articles that report mental health outcomes are not necessarily authored by mental health practitioners trained to use the DSM to diagnose specific mental health disorders, we anticipate that journal articles will use broad classifications (e.g. depression or anxiety) when reporting mental health impacts. So long as these impacts were measured, we will accept broad or DSM-5^TM^-detailed classifications. Where possible, the impact of the disorder on adolescents’ daily functioning (e.g. school attendance or capacity to be socially engaged) will be noted too. No secondary outcomes will be considered.

### Risk of bias in individual studies

Two review authors will independently assess the risk of bias in the included quantitative studies using the Scottish Intercollegiate Guidelines Network (SIGN) methodology checklist [37]. This checklist includes 14 items, of which 12 assess research biases, including in selection, performance, attrition, and detection. The two review authors will evaluate these 12 items on all included studies and will use the Cochrane Collaboration Review Manager (RevMan) [38] risk-of-bias graph to present this information.

Disagreements between review authors over the risk of bias in individual studies will be resolved by discussion, with involvement of two additional review authors where necessary. Following Hughes-Morley et al. [39], we will not omit any studies that demonstrate bias or limited quality. Instead, we will clearly identify the biases of these studies using the RevMan risk-of-bias graph [38] and will de-emphasize the results in our synthesis.

### Data synthesis

Because we anticipate that studies will not be sufficiently homogenous to accommodate meta-analyses, the results will be tabulated and narratively synthesized. We will conduct our data synthesis according to the Synthesis Without Meta-analysis (SWiM) guidelines from Campbell et al. [40]. In line with SWiM guidelines, we will include a table explaining and outlining the reporting of our data synthesis. An advantage of narrative syntheses is their capacity to provide a detailed response to the question directing the review [41]. This should yield a detailed account of what is currently known about how pollution relates to adolescent mental health worldwide, as well as what facilitates adolescent mental health resilience in the face or aftermath of pollution exposure. This account will be useful to signpost limitations and silences in current understandings of adolescent mental health during/following exposure to pollution and to advocate for specific research and practice agendas. To ensure replicability of the narrative synthesis, we will make public the completed data-charting forms that informed the synthesis (e.g. as supplemental, online files when the review is published). LT and KH will lead the synthesis, with input from the remaining reviewers.

### Meta-biases

Meta-bias includes both the selective reporting of outcomes due to their significance, magnitude, or direction and publication bias [31]. To assess meta-biases due to selective outcome reporting, we will (1) evaluate whether studies have associated protocols and whether those protocols were published prior to the recruitment of participants; (2) look for discrepancies between the published article and protocol (for those studies with a protocol); and (3) contact authors of the study, where additional information is needed. We do not plan any assessment of meta-biases due to publication bias.

### Confidence in cumulative evidence

To assess confidence, we will apply the Grading of Recommendations Assessment, Development and Evaluation (GRADE [42]), which is a widely used and transparent framework for summarizing confidence in evidence presented. GRADE involves a separation between judgements of quality of the evidence and the strength of the recommendations. Quality of evidence is classified into high, moderate, low, and very low, with these evaluations based on the type of study conducted, limitations of the study, inconsistencies in results, indirectness of evidence, imprecision, and potential reporting bias. Strength of the recommendation is classified into strong and weak, based on the quality of the evidence, uncertainty about the effects, variability in values, and uncertainty about resource use (in the case of interventions).

## Discussion

If changes are needed during the course of conducting the systematic review, we will make a dated amendment to our published PROSPERO review protocol (CRD42020176664). Although we will not limit the language of publications, we anticipate that publications in languages other than Afrikaans, Cantonese, English, Hebrew, Mandarin, Portuguese, or Spanish will require the use of professional translation services as the reviewer team is familiar with the aforementioned languages only. Accurate reporting of the original content will hinge on the accuracy of such translation. Another potential limitation relates to the level of publication detail, particularly regarding exposures and mental health impacts. A lack of detail is likely to limit the usefulness of the review to policy makers and mental health practitioners. We intend to make these stakeholders (and others, including adolescent clients/patients, their families, caregivers, and scientists) aware of review findings through social media posts, presentations at international meetings, and an academic publication in a peer-reviewed journal.

Despite these possible limitations, we believe that the proposed review is overdue. Adolescents comprise at least 16% of the world’s population [43]. Pollution effects are threatening the wellbeing of this sizeable population [1]. To better ensure the transition to healthy adulthood and protect the wellbeing of the world’s current and future adolescents, a thorough understanding is needed of how adolescent mental health is affected by pollution and what might enable resilience to pollution effects. The review that we propose is a first step in gaining that thorough understanding.

## Supplementary Information

**Additional file 1.** PRISMA-P 2015 Checklist. This checklist has been adapted for use with protocol submissions to *Systematic Reviews* from Table 3 in Moher D et al: Preferred reporting items for systematic review and meta-analysis protocols (PRISMA-P) 2015 statement. *Systematic Reviews* 2015 4:1

**Additional file 2.** APA PsycArticles search strategy, modified as needed for other electronic databases

## Data Availability

Data sharing is not applicable—no new data were generated for the purposes of formulating this protocol.

## Abbreviations

DSM-5^TR^: Diagnostic and Statistical Manual of Mental Disorders
GRADE: Grading of Recommendations Assessment, Development and Evaluation
LMICs: Low- and middle-income countries
