# Supplementary material for: Effects of pollution on adolescent mental health: a systematic review protocol
Source: Syst Rev. 2021 Mar 27;10:85. doi: 10.1186/s13643-021-01639-z (PMC8005223; doi:10.1186/s13643-021-01639-z)
Supplement: Supplementary file 2 — Additional file 2. APA PsycArticles search strategy, modified as needed for other electronic databases [file 13643_2021_1639_MOESM2_ESM.docx]

**Supplemental File 2: APA PsycArticles search strategy, modified as needed for other electronic databases**

| Search terms:  pollut* or pesticid* or plasticizer* or metal* or DDT or solvent*  AND  mental health or mental illness or mental disorder* or psychiatric illness or psychiatric disorder* or psychiatric condition* or mood disorder* or anxiety-like or depressive-like  AND  foetus* or fetus or child* or adolescen* or youth* or teenager* or juvenil* or neonat* or young or gestation*or prenatal or larva* or embryonic  NOT  Heavy metal music  Delimiters: Scholarly (peer reviewed) journals; Language: Any |
| --- |
